# Daily School Physical Activity from before to after Puberty Improves Bone Mass and a Musculoskeletal Composite Risk Score for Fracture

**DOI:** 10.3390/sports8040040

**Published:** 2020-03-28

**Authors:** Felix Cronholm, Erik Lindgren, Björn E. Rosengren, Magnus Dencker, Caroline Karlsson, Magnus K. Karlsson

**Affiliations:** 1 Clinical and Molecular Osteoporosis Research Unit, Department of Orthopedics and Clinical Sciences, Skane University Hospital, Lund University, SE-205 02 Malmo, Sweden; felix.cronholm@med.lu.se (F.C.); Erik_lind05@hotmail.com (E.L.); bjorn.rosengren@med.lu.se (B.E.R.); caroline.i.karlsson@telia.com (C.K.); 2 Department of Physiology and Clinical Sciences, Skane University Hospital, Lund University, SE-205 02 Malmo, Sweden; Magnus.Dencker@skane.se

**Keywords:** bone mineral content, bone mineral density, bone size, boys, children, exercise, girls, muscle strength, muscle mass, physical activity, puberty, Tanner stage

## Abstract

This 7.5-year prospective controlled exercise intervention study assessed if daily school physical activity (PA), from before to after puberty, improved musculoskeletal traits. There were 63 boys and 34 girls in the intervention group (40 min PA/day), and 26 boys and 17 girls in the control group (60 min PA/week). We measured musculoskeletal traits at the start and end of the study. The overall musculoskeletal effect of PA was also estimated by a composite score (mean Z-score of the lumbar spine bone mineral content (BMC), bone area (BA), total body lean mass (TBLM), calcaneal ultrasound (speed of sound (SOS)), and muscle strength (knee flexion peak torque)). We used analyses of covariance (ANCOVA) for group comparisons. Compared to the gender-matched control group, intervention boys reached higher gains in BMC, BA, muscle strength, as well as in the composite score, and intervention girls higher gains in BMC, BA, SOS, as well as in the composite score (all *p* < 0.05, respectively). Our small sample study indicates that a daily school-based PA intervention program from Tanner stage 1 to 5 in both sexes is associated with greater bone mineral accrual, greater gain in bone size, and a greater gain in a musculoskeletal composite score for fractures.

## 1. Introduction

Thirty percent of children suffer a fracture before the age of 18 [1] and 50% of women and 22% of men after the age of 50 [2]. This results in enormous costs for society [3], costs that have to be reduced. One strategy could be to reduce risk factors for fracture [4,5,6,7]. Regular physical activity (PA) may be such a factor, as regular PA is associated with benefits in bone mass, neuromuscular function, and muscle strength, traits associated with fracture risk [8,9,10,11]. PA during growth is also associated with a gradually reduced fracture incidence [10], as well as a low fracture incidence in adulthood [12,13,14,15]. However, it is debated whether a population-based PA intervention program in children can achieve the same benefits.

There is no PA intervention study published that has followed the development of musculoskeletal traits from before to after puberty [10]. Such research is necessary as the pre- and early pubertal period is the period when 25% of adult bone mass is acquired [16]. Furthermore, since fracture risk not only depends on a single trait, a musculoskeletal composite score may better estimate the expected fracture protective effect of PA than the measurement of a single trait [17], similar to the use of a composite risk score when predicting cardiovascular events [18] or by the fracture risk assessment tool (FRAX) for fracture risk in the elderly [19,20]. No such composite score exists for fracture risk evaluation in children. The aim of this study was to evaluate if a daily school-based PA intervention program from before to after puberty induced beneficial gains in musculoskeletal traits and a musculoskeletal composite score for fracture.

## 2. Materials and Methods

The Pediatric Osteoporosis Prevention (POP) study is a population-based prospective controlled intervention study with the primary aim of investigating whether daily school-based PA improves musculoskeletal development and reduces fracture risk; the study design is described in detail in previous publications [10,11,21,22]. The POP cohort includes children from four government-funded and community-based elementary schools, all located in the same city and with similar socioeconomic status. Before the start of the study, all schools had the same amount of PA (60 min/week). We then assigned the first school as the intervention school, and the remaining three as control schools. We increased the amount of PA in the intervention school to 200 min/week, provided as daily classes of 40 min, all school days during all nine compulsory school years. The PA included moderate to intense activities from the regular PA curriculum, such as gymnastics, team sports, dancing, running, jumping, and playing activities. We had no registration as regard the proportion of different activities that were included in the curricula or proportion of impact and endurance exercise. Furthermore, we had no registration to what extent the children participated in the PA lessons, but PA classes are mandatory in Sweden. The control schools continued with the national standard of 60 min/week PA. The same teachers as before the study start led the PA classes in all schools. In the latest bone mass and muscle strength follow-ups of the pediatric osteoporosis prevention (POP) study, 40% of the children were still in pubertal development [10,23].

All children, 98% of Caucasian ethnicity, who started 1st grade during 1998–2000 in the four schools were invited, when they were 6–9 years old and in Tanner stage 1 [23]. Informed consent was obtained from the parents of 217 children (123 boys and 94 girls) in the intervention and 132 (68 boys and 64 girls) in the control schools. During the intervention period, 26 boys and 27 girls in the intervention, and 31 boys and 24 girls in the control schools left the study. We excluded 30 boys and 31 girls in the intervention and 11 boys and 22 girls in the control schools, who, at the last follow-up in 9th grade (final compulsory school year in Sweden), had not reached Tanner stage 5 [23]. We further excluded 4 boys and 2 girls in the intervention school and 1 girl in the control school, who had a chronic disease or medication that could interfere with bone growth.

At baseline and follow-up, we undertook several measurements: Bone mineral content (BMC; g) and areal bone mineral density (aBMD; g/cm^2^) for total body less head, left femoral neck (FN), and first to fourth lumbar spine vertebra (L1L4); bone area (BA; cm^2^) for FN and L1L4; body composition (BC) as total body lean mass (kg), with dual energy X-ray absorptiometry (DXA, DPX-L® version 1.3z, Lunar Corporation, Madison, WI, USA) [10,24,25]; calcaneal speed of sound (SOS; m/s), a measurement also used to estimate bone quality [26] by quantitative ultrasound (QUS, Lunar Achilles model 1061®, Lunar Corporation, Madison, WI, USA); and muscle strength (concentric isokinetic peak torque (PT; Nm) for right knee flexion (flex) at a speed of 60 and 180 degrees/sby a computerized dynamometer (Biodex System III Pro®, Biodex Medical Systems Inc. Shirley, NY, USA). We used the highest PT value of five repeated movements of flexion [2,10,24]. Dedicated research technicians performed all measurements and calibrated the DXA apparatus daily using a phantom. There was no long-term drift in the equipment. The coefficient of variation (%), evaluated by duplicate measurements in 13 healthy children, was 1.4%–5.2% for BMC, 1.5% for BA, 2.4%–2.6% for aBMD 0.2% for SOS, 6.7% for PT_flex60_, and 9.1% for PT_flex180_.

We measured body height (cm) with a Holtain Stadiometer (Holtain LTD, Pembrokeshire, UK) and body mass (kg) with an electric scale (Avery Berkel HL 120 Electric Scale, Avery Berkel, West Midlands, UK). Body mass index (BMI) was calculated as mass/height^2^. A research nurse assessed the Tanner stage [23] at baseline while self-assessment was used at the follow-up. Lifestyle (dairy intake, alcohol, smoking, medical conditions, medication use, duration of weekly organized leisure-time PA) was evaluated through a non-validated questionnaire with assistance from parents [10,22,24,25]. We calculated total PA as the sum of PA in school and organized leisure-time PA. For each individual, we then summarized the duration of PA at baseline, after half the study period and at follow-up, and then divided this sum by 3 to reach an estimate of the average duration of PA during the entire study period for each child.

Using the compulsory Swedish 1st grade school health examinations, we compared height, weight, and BMI between the children who accepted participation and those that refused and were ten unable to identify any statistically significant differences between the groups [27]. We were also unable to identify any statistically significant group differences at baseline in age, height, weight, BMI, PA, BMC, BA, aBMD, BC, SOS, and PT between children that remained in the study and those who left the study during the follow-up period (data not shown).

We used IBM SPSS Statistics® version 23 for all statistical analyses. We present data as absolute numbers (n), proportions (%), means with standard deviations (SD), or mean differences with 95% confidence intervals (95% CI). We calculated study period changes as the follow-up value minus the baseline value. The composite score was calculated as the mean Z-score (the number of SDs above or below the age and gender-predicted mean value) of L1L4 BMC, L1L4 BA, BC, SOS, and PT_flex180,_ with all traits associated with fracture risk [4,5,6,7,10,12,15,22,23,24,25,28,29], a score that has been shown to predict fractures in old men [20]. For group differences in trait changes, we used analysis of covariance (ANCOVA) adjusted for age at follow-up and the baseline trait value (for the composite score, only the baseline trait since Z-scores include the adjustment for age). We used Spearman’s correlation test to estimate the correlation between the average duration of PA during the study period and composite score changes. We regarded *p* < 0.05 as a statistically significant difference. All participants in the POP study and the parents or guardians provided written consent, the Ethics Committee of Lund University, Sweden (LU 453-98; 1998-09-15) approved the study, and the study is registered as a clinical trial (ClinicalTrials.gov.NCT000633828).

## 3. Results

We present sex-specific group characteristics in Table 1 and Table 2. Twenty-six children (18.6%) were, according to isoBMI, classified as being overweight and 7 (5.0%) as being obese. The only baseline group differences in musculoskeletal traits between the intervention and control groups were higher PT_flex180_ (*p* = 0.02) in the intervention boys and higher PT_flex60_ (*p* < 0.05) in the intervention girls (Table 2).

Boys in the intervention group during the study period gained higher L1L4 BMC (*p* = 0.02), L1L4 aBMD (*p* = 0.03), L1L4 BA (*p* = 0.03), and PT_flex180_ (*p* = 0.008) than boys in the control group (Table 3). Girls in the intervention group gained higher BMC at all measured sites (*p* = 0.003 to 0.03), aBMD for total body less head (*p* = 0.004) and L1L4 (*p* = 0.002), BA for L1L4 and FN (both *p* = 0.03), and calcaneal SOS (*p* = 0.003) than girls in the control group (Table 3). 

Both boys and girls in the intervention group gained favorable musculoskeletal composite scores than their sex-specific control groups (both *p* = 0.02). There was a correlation between the average duration of PA per week during the study period and study period changes in the composite score (R = 0.17; *p* = 0.04).

## 4. Discussion

Puberty is the period in which 25% of the adult bone mass is acquired [16], and the greatest skeletal response to mechanical load occurs during pre- and early puberty [30]. In light of this, it seems reasonable that PA-induced musculoskeletal effects should be monitored from before to after puberty. We found in this small cohort, where we followed children from Tanner stage 1 to stage 5, that both boys and girls with daily school PA achieved greater gains in bone mass, bone size, and a musculoskeletal composite score for fractures than boys and girls with school PA 1–2 times per week. The current data further provide a plausible mechanism for the previously reported inverse correlation between the number of years with daily school PA and low annual fracture incident rate ratio (IRR) [10,21]. Since PA-induced bone mass benefits in young years seem to be retained in adulthood [12,13,14,15], and children with high level of PA have a lower fracture incidence in adulthood [12,13,15], it is imperative that we continue to follow the POP cohort to evaluate if increased PA in school really is a strategy to reduce the adult fracture burden.

This study also highlights the difficulties encountered when evaluating the effect of a PA intervention by one single trait. If we had used femoral neck aBMD as a single endpoint variable, we would erroneously have concluded that our intervention was without effect. We addressed this difficulty by the use of a composite score. Finding a dose–response relationship between the weekly duration of PA and gain in the score, in addition with knowledge that the score predict fractures in old men [20], strengthens the view that this is a clinically relevant score. 

Randomized controlled trials (RCTs) have found beneficial bone mass effects by increased PA in children [10,11], and also bone morphology is influenced by PA in a beneficial way [10,11,15,31,32,33,34,35,36]. However, most published intervention studies include volunteers, and few have followed the PA effect beyond 24 months [10,11]. The most recent publication from the POP cohort found beneficial bone mineral accrual in girls but not boys by increased PA [10]. The problem with that study was that around 40% of all children had still not reached Tanner stage 5 [10]. Our current study supports that a school PA program throughout puberty results in measurable effects on bone mass and bone size. Such programs should probably be initiated before puberty [30,37,38], but no study has so far been able to identify the best age to start such interventions. Of great interest is also that children in school-based PA programs continue to have a high duration of PA not only during the intervention period [39] but also after [40]. This could be one reason why PA-induced high bone mass in childhood is also associated with high bone mass in adulthood [12,13,14,15].

The study’s strengths include the prospective, controlled, and population-based study design, that this is the only PA intervention study that has followed children from Tanner stage 1 to 5 and that this is the first study that has utilized a composite score to estimate the overall musculoskeletal effect of PA in children. Study limitations include the low participation rates, especially in the control group, and the high dropout frequency. This makes it questionable to generalize the results to the broader community based on this study alone. However, the two dropout analyses found no indication of selection bias. We also acknowledge that most children in this study were of Caucasian ethnicity and they lived in a socioeconomic middle class area, facts that makes it difficult to transfer the inferences to children with other ethnic backgrounds and children living in other socioeconomic areas. The lack of individual randomization is another weakness. Further limitations include that we only registered the self-reported duration of school PA and organized PA during leisure time, with no type of activity, playing activities, or objectively registered PA being measured.

## 5. Conclusions

In our small sample study, there are indications that a daily school-based PA intervention program from Tanner stage 1 to 5, in both sexes, is associated with greater bone mineral accrual, greater gain in bone size, and a greater gain in a musculoskeletal composite score for fractures. Future larger studies, also in other socioeconomic settings and within other ethnic subgroups, should verify or oppose our findings before generalization of results to the broader community is possible. In addition, even if the composite score predicts fractures in old men, future studies must, as there may be different risk factors for fractures in old and young individuals, evaluate if the score predicts fractures in children as well.

## Figures and Tables

**Table 1:** Anthropometry, lifestyle characteristics, and duration of organized physical activity (PA). Data are presented as numbers (n) with proportions (%) within brackets or as means with standard deviations (SD) within brackets.

	Boys		Girls	
	Intervention (n = 63)	Control (n = 26)	Intervention (n = 34)	Control (n = 17)
**Before intervention start**				
Total organized PA (hours/week)	3.1 (3.5)	3.4 (3.4)	1.6 (1.7)	1.9 (1.6)
**After intervention start**				
Age (years)	7.6 (0.6)	8.2 (0.6)	7.5 (0.4)	8.0 (0.6)
Height (cm)	128.7 (6.0)	131.5 (5.5)	127.8 (5.6)	130.9 (7.5)
Weight (kg)	28.2 (5.3)	29.0 (5.0)	28.6 (5.8)	27.5 (5.2)
BMI (kg/m^2^)	16.9 (2.4)	16.7 (2.0)	17.4 (2.8)	16.0 (1.8)
Exclusion of dairy products	0 (0%)	4 (16%)	0 (0%)	0 (0%)
Chronic medical conditions	9 (14%)	2 (8%)	5 (15%)	0 (0%)
Current medication	13 (21%)	2 (8%)	5 (15%)	0 (0%)
Total organized PA (hours/week)	6.3 (3.5)	4.2 (3.4)	4.9 (1.7)	2.7 (1.6)
**Follow-up**				
Age (years)	15.2 (0.4)	15.3 (0.5)	15.1 (0.5)	15.3 (0.5)
Height (cm)	175.6 (6.9)	175.8 (6.7)	166.6 (5.0)	168.4 (6.0)
Weight (kg)	66.0 (11.3)	64.7 (10.7)	62.5 (10.1)	56.6 (8.6)
BMI (kg/m^2^)	21.4 (3.2)	20.9 (3.0)	22.5 (3.1)	19.7 (2.5)
Smoking	3 (5%)	1 (4%)	3 (9%)	4 (24%)
Drinking alcohol	15 (24%)	2 (8%)	6 (18%)	3 (18%)
Total organized PA (hours/week)	9.5 (4.9)	6.4 (3.2)	7.7 (3.2)	5.0 (3.2)

**Table 2:** Single musculoskeletal traits and a musculoskeletal composite score at baseline. Data are presented as numbers (n), means (standard deviations), or mean differences with 95% confidence intervals in brackets. BC = body composition, BMC = bone mineral content, aBMD = areal bone mineral density, BA = bone area, QUS = quantitative ultrasound, PT = muscle peak torque. QUS data were missing in 5 intervention and 16 control children.

	Boys			Girls		
	Intervention (n = 63)	Control (n = 26)	Mean Difference	Intervention (n = 34)	Control (n = 17)	Mean Difference
**BC (kg)**						
Lean mass	21.8 (2.6)	22.5 (2.7)	−0.6 (−1.9, 0.6)	19.8 (2.5)	20.6 (2.6)	−0.8 (−2.4, 0.7)
**BMC (g)**						
Total body less head	673.1 (144.9)	712.6 (141.8)	−39.5 (−106.2, 27.2)	638.4 (143.9)	644.9 (137.8)	−6.5 (−93.5, 80.5)
L1L4	19.9 (4.5)	21.2 (4.4)	−1.2 (−3.3, 0.8)	19.5 (4.8)	19.2 (3.5)	0.3 (−2.3, 3.0)
Femoral neck	2.9 (0.6)	3.0 (0.4)	−0.1 (−0.3, 0.2)	2.6 (0.5)	2.4 (0.3)	0.2 (−0.1, 0.5)
**aBMD (g/cm^2^)**						
Total body less head	0.70 (0.05)	0.71 (0.05)	−0.01 (−0.04, 0.01)	0.69 (0.05)	0.69 (0.05)	0.00 (−0.03, 0.03)
L1L4	0.67 (0.11)	0.69 (0.07)	−0.02 (−0.07, 0.02)	0.69 (0.12)	0.66 (0.07)	0.03 (−0.03, 0.10)
Femoral neck	0.80 (0.10)	0.81 (0.11)	−0.01 (−0.06, 0.04)	0.72 (0.10)	0.68 (0.06)	0.04 (−0.01, 0.09)
**BA (cm^2^)**						
L1L4	29.6 (3.4)	30.3 (3.4)	−0.8 (−2.3, 0.8)	27.8 (3.4)	29.0 (3.5)	−1.2 (−3.2, 0.9)
Femoral neck	3.6 (0.4)	3.7 (0.3)	−0.1 (−0.2, 0.1)	3.6 (0.3)	3.6 (0.4)	0.0 (−0.2, 0.2)
**QUS**						
SOS (m/s)	1533.6 (23.4)	1532.6 (18.4)	1.0 (−12.0, 13.9)	1525.3 (17.5)	1522.7 (15.9)	2.6 (−9.1, 14.3)
**PT (Nm)**						
PTflex60	23.7 (6.5)	25.9 (7.0)	−2.2 (−5.3, 0.9)	21.1 (5.7)	24.5 (5.3)	−3.4 (−6.7, −0.1)
PTflex180	21.5 (5.5)	24.7 (6.6)	−3.2 (−5.9, −0.5)	19.1 (6.3)	22.1 (4.5)	−3.1 (−6.6, 0.4)
**Composite score**	−0.07 (0.70)	0	−0.06 (−0.40, 0.28)	0.23 (1.25)	0	0.24 (−0.33, 0.81)

**Table 3:** Musculoskeletal trait and composite score changes. Data are presented as absolute numbers (n), means (standard deviations), or mean differences with 95% confidence intervals in brackets. BC = body composition, BMC = bone mineral content, aBMD = areal bone mineral density, BA = bone area, QUS = quantitative ultrasound, PT = muscle peak torque. Mean follow-up time was 7.5 years. ^a^ Analysis (ANCOVA) adjusted for age at follow-up and baseline trait value. QUS data were missing in 20 intervention and 17 control children.

	Boys				Girls			
	Intervention (n = 63)	Control (n = 26)	Mean Difference ^a^	*p* Value ^a^	Intervention (n = 34)	Control (n = 17)	Mean Difference ^a^	*p* Value ^a^
**BC (kg)**								
Lean mass	29.6 (4.8)	28.8 (4.2)	1.2 (−0.8, 3.3)	0.24	18.6 (2.8)	18.3 (3.4)	0.6 (−1.2, 2.5)	0.49
**BMC (g)**								
Total body less head	1710.5 (352.4)	1666.9 (386.1)	96.1 (−58.9, 251.1)	0.22	1437.9 (295.5)	1287.5 (252.1)	171.0 (31.1, 310.9)	0.02
L1L4	43.2 (10.3)	39.9 (11.8)	5.3 (1.0, 9.6)	0.02	41.6 (9.4)	35.3 (7.4)	6.6 (2.3, 10.9)	0.003
Femoral neck	3.1 (0.7)	2.9 (0.9)	0.1 (−0.2, 0.5)	0.44	2.8 (0.8)	2.2 (0.7)	0.6 (0.1, 1.1)	0.03
**aBMD (g/cm^2^)**								
Total body less head	0.37 (0.07)	0.36 (0.08)	0.02 (−0.02, 0.05)	0.30	0.35 (0.06)	0.30 (0.05)	0.05 (0.02, 0.08)	0.004
L1L4	0.43 (0.10)	0.39 (0.10)	0.05 (0.01, 0.10)	0.03	0.50 (0.11)	0.41 (0.08)	0.10 (0.04, 0.16)	0.002
Femoral neck	0.28 (0.10)	0.27 (0.13)	0.02 (−0.04, 0.07)	0.52	0.34 (0.10)	0.30 (0.11)	0.05 (−0.02, 0.11)	0.17
**BA (cm^2^)**								
L1L4	27.4 (4.4)	25.7 (4.7)	2.2 (0.2, 4.1)	0.03	23.1 (3.1)	21.7 (2.8)	1.9 (0.2, 3.6)	0.03
Femoral neck	1.9 (0.4)	1.8 (0.5)	0.0 (−0.2, 0.2)	0.80	1.5 (0.5)	1.2 (0.5)	0.3 (0.0, 0.6)	0.03
**QUS**								
SOS (m/s)	61.1 (39.4)	72.8 (37.9)	−10.1 (−32.3, 12.1)	0.37	86.4 (35.7)	42.5 (24.9)	41.4 (15.6, 67.3)	0.003
**PT (Nm)**								
PTflex60	68.4 (21.1)	66.2 (16.4)	2.9 (−6.6, 12.4)	0.55	44.3 (11.3)	38.3 (13.3)	7.7 (−0.2, 15.7)	0.06
PTflex180	51.5 (13.4)	43.7 (9.9)	8.2 (2.2, 14.2)	0.008	30.7 (8.8)	25.8 (9.5)	4.6 (−1.4, 10.5)	0.13
**Composite score**	0.30 (0.55)	0 (1)	0.29 (0.05, 0.54)	0.02	0.31 (0.88)	0 (1)	0.43 (0.08, 0.78)	0.02
